# Perceptions, barriers, and coping preferences towards quitting among ethnic minority young adults with alcohol use disorder: a phenomenological study

**DOI:** 10.3389/fpubh.2025.1593064

**Published:** 2025-09-18

**Authors:** Getaneh Mulualem Belay, Yim Wah Mak, Ka Yan Ho, Katherine Ka Wai Lam, Qi Liu, Funa Yang, Ting Mao, Xi Chen, Ling Jiang

**Affiliations:** School of Nursing, Hong Kong Polytechnic University, Kowloon, Hong Kong SAR, China

**Keywords:** alcohol use disorder, barrier, ethnic minorities, perception, quitting

## Abstract

**Introduction:**

Alcohol use disorder (AUD) significantly impacts the lives of young adults from ethnic minority groups. Understanding their experiences with quitting is essential for developing culturally specific interventions. Therefore, this study aimed to explore the perceptions, barriers, and coping strategies related to quitting among young ethnic minority adults with AUD.

**Method:**

A descriptive phenomenology study was conducted. The sample size of 22 was determined by achieving data saturation, the point at which no additional themes were identified in the analysis. Interviews were audio-recorded, transcribed, and analyzed using Colaizzi’s method.

**Results:**

A total of 22 participants were interviewed. Themes related to perceptions included: (1) alcohol use is normal; (2) quitting is good for health but difficult, and (3) Quitting is nice but not now. The study also identified four barriers including separation from family, withdrawal symptoms, peer influences, and challenges arising from acculturation. Coping preferences highlighted in the study included distraction, exercise, counseling, and reuniting with family.

**Conclusion:**

The findings underscore the complex interplay of cultural, social, and personal factors in the quitting process and emphasize the need for culturally tailored interventions. Mental health nurses should pay special attention to ethically marginalized and discriminated populations. These findings can also guide nurses in considering the cultural context, barriers, and perceptions of ethnic minorities in quitting, thereby enhancing culturally competent care.

## 1 Introduction

Alcohol use disorder (AUD) is a severe medical condition characterized by the inability to stop or limit alcohol use, despite negative consequences (1). AUD is a major global health concern, more prevalent in developed countries due to alcohol’s affordability, accessibility, and aggressive marketing (1). All populations are at risk of developing AUD, despite variations in its prevalence across age, race, gender, and culture (2–4). Recent evidence suggests that young ethnic minority populations are highly vulnerable to substance use, including alcohol (2, 5, 6).

Unlike the local populations, young people from ethnically diverse communities are profoundly influenced by AUD (6–8). They encountered stigma, discrimination, and exclusion from various activities, which led to acculturative stress, depression, isolation, and life dissatisfaction, driving them to use alcohol as a coping mechanism (8–10). Research on ethnic minorities indicated that the prevalence of lifetime AUD was 21% (5), which concurred with previous data that revealed 21% of college students, 19% of non-students, 15% of part-time college students, and 12% of non-college students (11).

Despite the high prevalence of AUD, ethnic minority young people addicted to alcohol remained untreated (7, 11). Evidence indicated that only 4% of ethnic minority groups received treatment for AUD (11). This could be due to a lack of culturally tailored treatment, language barriers, bias, discrimination, and a lack of proper support (7). Apart from this, evidence from prior research revealed that ethnic minorities had a limited understanding of AUD consequences on their health, education, and economic activities (12–15).

Quitting is the key option to reduce the impact of AUD. However, it remains a significant challenge, due to the addictive nature of alcohol. Recent research showed the success rate of quitting was not promising (16, 17). One study found only 10–18% of alcohol abusers/dependents were trying to quit, and of those, only 30–38% successfully quit (16).

Concerning factors affecting quitting, some quantitative studies have shown that female gender, being married, and older age promoted quitting (16). However, these studies were conducted in the general population. To date, there is little evidence about the perceptions and challenges towards quitting in ethnic minority young adults, even though they are generally in a less favorable position in accessing medical services than the general population (7, 18).

Previous interventional studies were not promising in ethnic minorities (17, 19, 20), possibly due to a lack of tailoring to their specific barriers. Even existing interventions for this population mainly focus on other addictions, such as smoking (21–23). In addition, previous quantitative studies assessed AUD prevalence and associated factors but overlooked their perspectives and quitting barriers (24). Understanding their perceptions, barriers, and coping strategies is crucial for developing appropriate interventions. To shed light on this literature gap, the current study aimed to examine the perceptions, barriers, and coping preferences toward quitting among ethnic minority young adults with AUD.

## 2 Materials and methods

### 2.1 Study design and setting

A descriptive phenomenology qualitative design was conducted in Hong Kong from March 1 to June 30, 2023.

### 2.2 Study participant selection

The study examined ethnic minority young adults with AUD in Hong Kong. This segment of the population often faced challenges such as social marginalization, limited access to services, and pressures of cultural adaptation. Snowball sampling was employed to effectively reach participants from this community. The first participant was recruited in an African Center, a non-profit organization where Africans gathered. The sample size of 22 was determined by achieving data saturation, the point at which no additional themes were identified in the analysis. This method aligns with qualitative research evidence, where saturation is typically reached within 15–30 interviews (25, 26). Although the sample size may seem small, it was sufficient to explore participants’ perceptions, barriers, and coping preferences for quitting. The collective experiences of this cultural group, along with the richness of qualitative data, provided valuable insights despite the limited sample size.

#### 2.2.1 Eligibility criteria

The following criteria were considered for inclusion: (1) young adults from Hong Kong’s ethnic minority groups who have lived in the city for at least 6 months; (2) they must be able to hear, read, speak, and understand conversations in English; (3) they must be legal residents of Hong Kong and aged from 17 to 30; (4) they must be able to give their consent; and (5) they must have AUD based on AUDIT score of 8 or above.

### 2.3 Data collection

Participants initially completed a sociodemographic and AUDIT questionnaire. Participants with AUDIT score ≥ 8 were asked to participate in individual semi-structured interviews at Tsim Sha Tsui, Kowloon Chung King Mansion. This location was chosen as it was a hub for multiple ethnic minority groups, making it convenient to reach the participants. The interviews were conducted by a male registered nurse and PhD candidate, who had learned about data collection from a senior qualitative researcher. The interviewer had no previous relationship with the participants, but he built a rapport with them before the interview. The interview was conducted using a semi-structured interview guide containing open-ended and flexible questions, developed by a qualitative study expert. The interviews lasted between 10 to 30 min. The four main topics covered were: (1) perceptions towards AUD and quitting, (2) barriers to quitting attempts, (3) quitting experiences, and (4) perceived coping preferences. All interviews were audio-recorded, and the interviewer noted nonverbal cues throughout the conversations.

### 2.4 Data analysis

Descriptive statistics were used to report the demographic characteristics of the participants. The recorded audio was then directly transcribed into English verbatim for further analysis. Initially, all qualitative data were coded and displayed in a table. Two authors regularly cross-checked the codes and themes developed based on the available data to verify that the codes and themes were correct and representative. They used Colaizzi’s method of analysis (27). Then, the results were reported following the Consolidated Criteria for Reporting Qualitative Research (COREQ) (28).

### 2.5 Research rigour

The same interviewer conducted the interviews to ensure reliability and credibility. Themes and codes were constantly compared to existing data to ensure they accurately reflected participants’ quitting experiences. The study team also used bracketing, a technique where the researcher suspends their preconceptions to view and explain the phenomena objectively (27). Besides, the findings of the study were validated by external experts.

### 2.6 Ethical considerations

Ethical approval was issued by the Institutional Review Board (IRB) of Hong Kong Polytechnic University (reference number: HSEARS20230109001). Before an interview, informed consent was obtained from each participant. All information obtained from face-to-face interviews with participants was kept non-identifiable and private. The confidentiality and privacy of the data were preserved throughout the study process.

## 3 Results

### 3.1 Sociodemographic characteristics

A total of 31 participants were invited for interviews, and 22 agreed to participate, with a response rate of 88%. The average age of participants was 24.7 (SD = 4.0) years, ranging from 17 to 30 years. As illustrated in Table 1, most of the participants were females (68.2%), catholic (59.1%), Filipino (50%), living alone (40.9%), single in marital status (77.3%), and domestic helpers (40.9%).

**Table 1:** Socio-demographic characteristics of ethnic minority young adults, 2024.

Variables	Number (%)
Sex	
Male	7 (31.8)
Female	15 (68.2)
Religion	
Catholic	13 (59.1)
Muslim	7 (31.8)
Christian	2 (9.0)
Living in Hong Kong	
<5 years	6 (27.3)
5–10 years	8 (36.4)
10–15 years	6 (27.3)
≥15 years	2 (9.1)
Ethnicity	
Filipino	11 (50)
Pakistan	1 (4.5)
African	10 (45.5)
Marital status	
Single	17 (77.3)
Married	3 (13.6)
Others	2 (9.1)
Occupation	
Sale worker	1 (4.5)
Homemaker	4 (18.2)
Daily laborer	2 (9.1)
Craftsperson	1 (4.5)
Student	5 (22.7)
Domestic helper	9 (40.9)
Educational status	
Below Primary	1 (4.5)
Junior secondary education	1 (4.5)
Senior Secondary	11 (50)
Post-secondary	9 (40.9)
Employment status	
Self-employed	5 (22.7)
Unemployed	3 (13.6)

### 3.2 Perceptions towards AUD and quitting

#### 3.2.1 Theme 1: alcohol use is normal

Many participants (*n* = 8) did not see their drinking as harmful and were unwilling to quit without a medical reason. They believed increased alcohol tolerance could lessen the impacts of AUD. The social acceptance of alcohol, its availability in events, and its perceived stress-relieving benefits made them view drinking as normal. During the semi-structured interview, a participant said, *“I do not see a reason to quit. I think maybe the only thing that would push me to give up is if my liver is not well, you know, … I’ll think about it, but otherwise, I do not see that happening, you know if it’s not killing me, if it is regulated, and I do not see the point of giving up”* (21-year-old male African).

#### 3.2.2 Theme 2: quitting is good for the health but difficult

Some participants (*n* = 10) were optimistic about quitting or reducing their alcohol use, as they perceived it would improve their health, productivity, income, and engagement in meaningful activities. However, they faced various stressors related to acculturation, discrimination, or socioeconomic challenges, which led them to use alcohol as a coping strategy. Quitting alcohol without addressing these underlying issues was challenging. For example, in his semi-structured interview, a participant said, *“I feel like I must quit. I want to quit but cannot quit. That’s how I feel. I cannot wait, but I feel I have one or two, and I must. But it is hard”* (29-year-old male African).

#### 3.2.3 Theme 3: quitting is nice but not now

Some participants (*n* = 3) acknowledged the need to quit but preferred to do so later, after achieving personal goals like owning a home/car, financial stability, marriage, or a better lifestyle. They felt they needed enjoyment and relaxation from drinking with friends while young and planned to quit only when they reached old age around 70–80 years old. For instance, a participant from Africa stated, *“I plan to quit when I reach my old age, you know. So, when I’m around 80 or 70 years old, God willing, if I live to that age, I could, yeah”* (29-year-old male African).

### 3.3 Barriers to quitting alcohol

#### 3.3.1 Theme 1: separation of families

Several participants (*n* = 5) mentioned that separation from family hindered their ability to quit. The family was perceived to provide emotional support, care, advice, and counseling that could help the quitting process. However, separation led to feelings of isolation, stress, loneliness, and vulnerability, making it difficult to resist urges and cravings to drink. For example, during her interview, a participant said, *“When I am in the Philippines, I am taking care of my kids, I have a husband, and when I separate them, back to drinking again, you know, sad. That’s why I drink because I need to forget”* (29-year-old female Filipino).

### 3.4 Theme 2: withdrawal symptoms

Some participants (*n* = 4) faced withdrawal symptoms like boredom, irritability, and physical discomfort when attempting to quit, which led to worry, fear, and anxiety that hindered their quitting efforts. This was exemplified by a Filipino participant who experienced headaches, shaking, and sleep difficulties while trying to quit, which ultimately led her to resume drinking. She said, “*When I stop drinking, sometimes I get a headache. Like, I cannot sleep also, because all my body needs to drink. This is a difficult assignment. But I also have my hand shaking like I want to grab it”* (27-year-old female Filipino). Similarly, a participant from Africa said, *“I found myself to be so lonely, so bored, and so lost. That I was like, I cannot live like this. No social activity, I feel lost, I feel empty with nothing to do. Yeah, no social activity. So, you know”* (29-year-old male African).

#### 3.4.1 Theme 3: Peer influence

Peer influence was a key barrier for participants (*n* = 6) in planning to quit. They feared losing friendships if they refused to drink invitations, so they prioritized social relationships over quitting. For instance, in the semi-structured interview, a participant said, *“If I refuse friends’ drinking invitations, other friends get jealous and angry. I have to go even though I do not want to, to avoid them getting upset with me”* (30-year-old female Filipino).

#### 3.4.2 Theme 4: challenges resulted from acculturation

The challenge resulting from acculturation was reported by some participants (*n* = 3) as their barrier to quitting. The cultural stigma, language barrier, being marginalized, and being excluded from jobs, school, social, and economic activities have significantly impacted their quitting process. For instance, one participant said, *“I’m so frightful for my daughter, who is not going to school, I’m not happy, Seriously, even drink it. I’ve been here 17 years but did not get my ID, and I’m doing work from Ghana people. They helped make payments. Trust me, you cannot get the motivation to do it. The government is not paying for your house”* (29-year-male-African).

### 3.5 Perceived coping strategies for quitting

#### 3.5.1 Theme1: distraction

Some participants (*n* = 3) said they would try to keep busy with activities like watching movies, chatting with friends, and playing video games to divert their attention from drinking. They preferred digital activities as they found them more convenient and entertaining, helping redirect their concentration away from cravings and triggers. For instance, one participant said, *“I always like to watch YouTube movies to divert myself. Divert yourself watching YouTube, playing games, and other activities that I usually do”* (24-year-old female Filipino). Similarly, another participant said, *“Yeah, focus on mobile and watch movies, roaming around some cities and take pictures to busy me, get busy with TV, and social media”* (25-year-old female Filipino).

#### 3.5.2 Theme 2: exercise

Several participants (*n* = 7) felt that exercise could enhance their chances of quitting successfully. They believed playing sports could help reduce stress, anxiety, and depression, which were major reasons for their drinking, so they would not need to rely on alcohol to cope. For instance, a participant said, *“Joining sports like Zumba or hiking with friends could help relieve tension, When I feel like quitting alcohol, I do hike, do sports something like that, yeah”* (30-year-female-Filipino).

#### 3.5.3 Theme 3: counseling

Participants (*n* = 3) said they required advice and support from friends and counselors to manage challenges during the quitting process, as they lacked guidance on dealing with issues in their studies and work, which contributed to their alcohol addiction. For example, a participant said, *“I think counseling from people who have struggled with and recovered from alcoholism would be helpful if they come out and explain the story like, what happened, and why quit. Hearing their real personal stories and experiences, would be helpful, and convince me to take the issue seriously and change my behavior, you know”* (21-year-male-African).

#### 3.5.4 Theme 4: reunion with family

Some participants mentioned that the support from family was crucial to motivate them to think about quitting and improve their chances of success. This is because they did not want to be shameful in front of their family members and be labeled as alcoholics by the family. For example, a participant stated, *“Actually; I drink alcohol again when I’m here in Hong Kong. But when I return or vacation in the Philippines, I never drink alcohol because I’m with my kid. And I do not want my kid to see me drinking because I do not want my kid to think that I’m an alcoholic mother”* (27-year-old female Filipino).

## 4 Discussion

AUD significantly affected the livelihoods of ethnic minority young adults in terms of their physical health, psychological and social functioning, and financial conditions. To mitigate these impacts, understanding their quitting experiences is crucial to guiding the development of culturally specific interventions. Therefore, this qualitative study examined the perceptions and barriers to quitting among Hong Kong ethnic minority young adults with AUD. In addition, we also explored their perceived coping strategies for quitting.

This study revealed that the perceptions towards quitting alcohol use were mixed in ethnic minority young adults. On one hand, they understood that quitting is favorable to their health and lives. On the other hand, they refused to take immediate action because of different reasons, including that they had to achieve more important personal goals, and quitting was difficult owing to the addictive nature of alcohol. Some ethnic minority young adults even did not recognize the harmful effects of alcohol drinking and the importance of quitting. Their normalization of alcohol use could be explained by two reasons. Firstly, they did not experience any serious medical issues associated with alcohol use, and hence there was no cue to action which is known to be a very important factor in motivating people for behavioral change in the Health Belief Model (29, 30). Secondly, they thought that continued drinking could build up their body’s tolerance to alcohol which in turn prevented them from the harmful effects of alcohol. This reflected those misconceptions towards alcohol use that existed in some ethnic minority young adults. These misconceptions might partly come from their culture and partly be related to their low educational levels; therefore, they were not well-educated on the health effects of alcohol.

This study revealed four different barriers to quitting for ethnic minority young adults. These barriers included family separation, challenges resulting from acculturation, peer influences, and withdrawal symptoms associated with alcohol quitting. Based on the first two barriers, we can deduce that acculturation is a critical period for ethnic minority young adults which makes them more vulnerable to developing AUD. As revealed by the semi-structured interviews, ethnic minority young adults have faced serious challenges in fitting themselves into the new environment. Language barriers, discrimination, and being marginalized led them to rely on alcohol as their coping strategy to manage their negative emotions. Therefore, despite being motivated to quit, they failed in the quitting process and relapsed back to alcohol use. Likewise, due to the separation of family, friends who met in the new environment became their primary source of social support. Nevertheless, these friends were also mainly ethics minorities who encountered similar challenges and relied on alcohol use as their coping strategy. This created a conducive environment for drinking and further reinforced the drinking behaviors in the ethnic minority group. Apart from the aforementioned issues that are related to acculturation, and withdrawal symptoms, a well-recognized barrier also impeded ethnic minority young adults from successfully quitting. In the qualitative interviews, ethnic minority young adults who made quit attempts experienced a lot of withdrawal symptoms, such as handshaking, severe headaches, feelings of loss, boredom, loneliness, and emptiness. This could be attributed to the psychological and physical effects of alcohol (31). One thing worth noting is that some participants mentioned that they were unable to receive any counseling and advice to support alcohol quitting from social services. This qualitative expression provided further support to illustrate the social exclusion experienced by ethnic minorities. In fact, numerous evidence has shown that counseling and advice from professionals are effective in helping people with AUD to manage withdrawal symptoms (32). To address the social exclusion and needs of ethnic minority young adults, the government should consider extending its existing services on AUD to cover this vulnerable population group.

This study also found four strategies that were perceived as useful by ethnic minority young adults to assist them in quitting. These strategies were a distraction, exercise, counseling, and reunion with family. Concerning distraction, the most reported methods by our participants included watching television and movies, playing video and mobile games, browsing websites via mobile phones and computers, and chatting with friends using different information communication technologies, e.g., WhatsApp and Facebook Messenger. Drawing on these qualitative expressions, it is observed that ethnic minority young adults were likely to make use of different technologies to distract themselves from alcohol craving and withdrawal symptoms. The preference for digital distraction could be attributed to the busy schedules of over two-thirds of our participants who were employed either full-time, part-time, or self-employed, making them to be difficult to seek face-to-face interventions from nearby health institutions. The language barriers between healthcare providers and ethnic minority young adults, as shown by a study conducted in Hong Kong, may also contribute to this preference [14]. These findings underscore the importance of establishing online interventions to address the needs of this segment of the population.

The second perceived action that appeared beneficial for quitting or reducing drinking was physical activity. As illustrated in the qualitative interviews, several participants recommended doing exercise and sports and engaging in other everyday physical activities, e.g., housework helped distract attention from drinking temptations and reduce acculturation stress and other negative emotions. These results also concurred with previous randomized controlled trials on people with AUD that exercise-based interventions were effective as a standalone treatment in promoting alcohol abstinence (33, 34) in which exercise can help regulate the dysfunctional reward system via facilitating the dopaminergic transmission, modifying the dopaminergic signaling, and introducing an alternative reward pathway (35). Given the effectiveness of physical activity in promoting alcohol abstinence in AUD and the high acceptability of such a method by ethnic minority young adults, physical activity could be considered as one of the interventional components or even as a standalone treatment for ethnic minority young adults with AUD. Lastly, several participants stated that counseling from counselors or friends and reconnecting with family members were helpful to assist them in abstaining from alcohol use during the quitting process. These treatment preferences were also related to one of their reasons for developing AUD, which is separation from their family members and their original friends (36, 37). Given the development of information communication technology, how to make use of this method to provide necessary social support for ethnic minority young adults with AUD during the quitting process is crucial.

## 5 Limitation

This study, despite its originality, has some limitations that need careful consideration. First, the relies on snowball sampling disproportionately recruited Filipino, African, and Pakistani, reducing generalizability to other ethnic minorities. Second, gender representation was skewed heavily toward females, reflecting Hong Kong’s domestic helper demographics. Lastly, AUD diagnosis relied solely on self-reported drinking metrics without biochemical validation (e.g., blood tests) or clinician assessment, which may raise concerns about the diagnosis.

## 6 Implications for further research

The study results suggested that ethnic minority young adults with AUD faced challenges when attempting to quit drinking. Conditions such as the addictive effects of alcohol, misconceptions of AUD and its consequences, unclear life goals, and reluctance to commit to sobriety significantly affected their efforts to quit. These findings highlighted the importance of developing appropriate interventions to strengthen their effort to quit. A systematic review and meta-analysis on the effectiveness of psychosocial intervention for AUD in young adults found that acceptance and commitment therapy, one of the third waves of behavioral therapies, maybe a potential treatment option (38–40). A previous qualitative study of Hong Kong ethnic minority young adults with AUD similarly supported the high relevancy of acceptance and commitment therapy to this population group as most of them started their drinking because of losing their life goals during acculturation. This qualitative study further identified important treatment components, including the use of digital technology, counseling, and social support. Future researchers should consider how these components can be integrated into acceptance and commitment therapy to develop an effective, feasible, and acceptable intervention for ethnic minority young adults with AUD.

## 7 Conclusion

This study explored the perceptions, barriers, and perceived coping strategies towards quitting among ethnic minority young adults with AUD. The results suggested that this population group was ambivalent towards quitting. They perceived that quitting was beneficial but was very difficult and not an immediate action for them if there was no medical indication. For those who attempted to quit, the addictive effect of alcohol, ongoing challenges in acculturation, and a lack of support from family members and friends made quitting difficult. Besides, the use of digital technology, counseling, and social support from family members and friends were important components that need to be considered when developing future interventions for this vulnerable group in society.

## Data Availability

The raw data supporting the conclusions of this article will be made available by the authors, without undue reservation.
